# The Effect of Structured Context on Chest Radiograph Interpretation by a Multimodal Large Language Model: A Pilot Comparative Study

**DOI:** 10.7759/cureus.108715

**Published:** 2026-05-12

**Authors:** Andrew Cusick, Shelby Guy, Christopher Herz, Colton Manfre, Rebecca DeVries

**Affiliations:** 1 Radiology, Rocky Vista University College of Osteopathic Medicine, Parker, USA; 2 Radiology, University of Nebraska Medical Center, Omaha, USA

**Keywords:** artificial intelligence in radiology, chatgpt, chest x-ray, deep learning artificial intelligence, msk radiology, musculoskeletal radiology

## Abstract

Background

Multimodal large language models are increasingly discussed as potential adjuncts for medical image interpretation, yet the extent to which structured contextual guidance affects performance remains uncertain.

Objective

The aim of this study was to determine whether a structured contextual intervention improves ChatGPT performance on chest radiograph interpretation.

Methods

We conducted a comparative pilot study using 50 chest radiographs with established reference diagnoses. In the baseline condition, ChatGPT interpreted each image using only the standardized prompt, “Diagnose this X-ray image.” In the structured-context condition, the model first reviewed a Radiopaedia-derived teaching module containing 100 labeled chest radiographs spanning 10 common diagnoses and then interpreted the same test set using an author-developed radiologic framework that emphasized study identification, image-quality assessment, systematic visual review, separation of findings from diagnostic impressions, explicit communication of uncertainty, and structured reporting. Performance was assessed with percent-correct scoring, with partial credit awarded when responses demonstrated appropriate reasoning but lacked full specificity.

Results

Overall accuracy improved from 28% at baseline to 62% after the structured-context intervention. Accuracy reached 100% for chronic obstructive pulmonary disease, pulmonary edema, and foreign body identification, and improved to 80% for pneumothorax, cardiomegaly, and pleural effusion. Performance remained limited for lung cancer (20%) and feeding tube placement (40%), and rib fractures were not identified in either condition. Accuracy for atelectasis declined from 40% to 20%.

Conclusions

Structured contextual guidance improved performance on selected chest radiograph tasks, but overall diagnostic reliability remained inadequate for independent clinical use. Larger studies with standardized scoring, external validation, and imaging-specific evaluation are needed.

## Introduction

Artificial intelligence (AI) is increasingly integrated into medical imaging workflows and is often viewed as particularly promising in radiology, where rapid analysis of complex visual data may support efficiency and clinical decision-making [1,2]. Interest in diagnostic AI has grown substantially as image-analysis tools have become more widely available [3,4]. Regulatory approvals have also increased, with radiology accounting for a substantial share of FDA-cleared AI and machine learning devices [5,6]. Despite this expansion, relatively few systems have been evaluated in rigorous clinical studies [5].

Traditional deep learning systems can achieve strong performance on selected radiographic tasks, but these models generally require large labeled datasets and task-specific training [1]. By contrast, large language models (LLMs) such as ChatGPT are general-purpose systems designed primarily for language reasoning and generation. Newer multimodal variants can also accept image inputs, making them relevant for exploratory assessment in medical imaging [7,8]. However, these models were not developed specifically for radiographic interpretation, and their performance characteristics in this setting remain uncertain.

One practical question is whether performance on image-interpretation tasks can be improved not through retraining but through structured contextual guidance that shapes attention, reasoning, and reporting. Radiographic interpretation depends not only on visual pattern recognition but also on a disciplined search strategy, careful separation of observed findings from inferred diagnoses, and explicit communication of uncertainty. It is therefore plausible that the context in which an LLM is asked to interpret an image could materially influence output quality [9,10].

This pilot study evaluated ChatGPT performance on chest radiograph interpretation under two conditions: (1) a baseline condition using only a minimal prompt and (2) a structured-context condition that combined labeled teaching examples with an author-developed radiologic interpretation framework. The objective was to determine whether structured contextual guidance could improve diagnostic performance without modifying model architecture or weights.

## Materials and methods

Methods

Study Design

This comparative pilot study evaluated the effect of structured contextual guidance on ChatGPT performance in chest radiograph interpretation. Model output was compared between a baseline condition without prior educational exposure or interpretive scaffolding and a structured-context condition, in which the model reviewed teaching material and then interpreted images using an author-developed radiologic framework.

Test Dataset

The test dataset consisted of 50 chest radiographs provided by Rebecca DeVries. Each image had an established reference diagnosis that served as the ground truth for evaluation. The dataset included pediatric, adult, and geriatric cases. Test images were not included in the educational dataset.

Educational Intervention

The educational intervention consisted of a PowerPoint-based teaching module derived from Radiopaedia [11]. The module included 100 labeled chest radiographs spanning 10 commonly encountered diagnoses, along with diagnosis labels, concise explanations of relevant radiographic features, and supporting demographic context. The 10 diagnostic categories were chronic obstructive pulmonary disease (COPD), lung cancer, pneumothorax, atelectasis, cardiomegaly, pleural effusion, foreign body, pulmonary edema, feeding tube placement, and rib fracture. This intervention was intended to simulate focused radiology instruction rather than formal model retraining.

Structured Interpretation

In the structured-context condition, the model also received an author-developed prompting framework designed to guide image analysis in a careful, systematic, and uncertainty-aware manner. The framework directed the model to identify the study type, body region, and completeness of the examination; comment on technical factors such as positioning, rotation, exposure, motion artifact, cropping, and absence of additional views when relevant; and then perform a structured review of the cardiomediastinal silhouette, lungs, pleura, focal opacities, edema patterns, pleural effusions or pneumothorax, osseous structures, soft tissues, and any lines, tubes, or devices. The model was further instructed to distinguish observed findings from inferred diagnoses, prioritize the interpretation, communicate uncertainty explicitly when image quality limited confidence, and flag findings that would require urgent in-person evaluation.

Evaluation Protocol

The same multimodal LLM and the same test image set were used in both evaluation conditions. In the baseline condition, each image was presented with the standardized prompt, “Diagnose this X-ray image,” without additional teaching examples or interpretive guidance. In the structured-context condition, the same prompt was used after the model had reviewed the teaching module and received the structured interpretation framework. Responses were recorded verbatim for analysis. No parameter-level retraining or architectural modification was performed.

Outcome Measures and Analysis

Model responses were compared with the established reference diagnoses. Diagnostic performance was quantified using percent-correct scoring, with partial credit assigned when the response reflected appropriate reasoning but lacked complete specificity. Accuracy was summarized descriptively for the baseline and structured-context conditions.

## Results

Overall diagnostic accuracy increased from 28% in the baseline condition to 62% after the structured-context intervention (Table 1). Baseline performance was lowest for COPD and rib fracture, both of which had 0% accuracy, and highest for pulmonary edema, which reached 60% accuracy. After the structured-context intervention, the largest gains were observed for COPD, pulmonary edema, and foreign body identification, each of which reached 100% accuracy. Performance also improved for pneumothorax, cardiomegaly, and pleural effusion, each reaching 80% accuracy after the intervention. In contrast, lung cancer remained at 20% accuracy, feeding tube placement remained limited at 40%, and rib fractures were not identified in either condition. Accuracy for atelectasis declined from 40% at baseline to 20% after the structured-context intervention. Taken together, these findings suggest that structured contextual guidance improved performance most clearly for abnormalities with relatively salient or pattern-based radiographic features.

**Table 1:** Diagnosis-specific diagnostic accuracy before and after the structured-context intervention COPD, chronic obstructive pulmonary disease

Diagnosis	Baseline, %	Structured Context, %
COPD	0	100
Lung cancer	20	20
Pneumothorax	20	80
Atelectasis	40	20
Cardiomegaly	20	80
Pleural effusion	40	80
Foreign body identification	40	100
Pulmonary edema	60	100
Feeding tube placement	40	40
Rib fracture	0	0
Overall accuracy	28	62

## Discussion

This pilot study found that structured contextual guidance substantially improved ChatGPT performance on chest radiograph interpretation, increasing overall accuracy from 28% to 62%. Although this level of performance remains inadequate for independent clinical use, the direction of effect is consistent with emerging literature showing that multimodal LLM performance in radiology is highly sensitive to prompt design, few-shot examples, and the availability of supporting context [9,12,13]. In a chest-radiograph study, few-shot prompting improved GPT-4V detection metrics relative to zero-shot prompting, although the model was still judged not ready for real world diagnostic use [12]. Similarly, Zhu et al. found that removing detailed clinical history reduced multimodal ChatGPT-4V diagnostic accuracy on radiologic tasks, reinforcing the idea that context itself can materially shape performance [13].

An important implication of these findings is that the observed improvement did not result from retraining. Instead, performance changed after exposure to labeled teaching examples and a structured interpretation framework. That framework imposed a radiologic workflow: identify the study, assess technical adequacy, perform a systematic review, separate observed findings from diagnostic impressions, and communicate uncertainty explicitly. Reviews of radiology-focused prompt engineering similarly emphasize that structured prompting and contextual scaffolding can meaningfully influence output quality, even though they do not eliminate hallucination, factual error, or other reliability concerns [9,10].

However, the 62% post-intervention accuracy observed here remains below the performance reported for systems developed specifically for chest radiograph interpretation. That comparison must be made cautiously because tasks, datasets, and metrics differ across studies; nonetheless, it highlights the gap between general-purpose multimodal models and domain-adapted imaging AI. For example, CXR-LLaVA was trained on large chest-radiograph datasets and achieved mean F1 scores of 0.81 on an internal test set and 0.56 on an external test set for six major findings, outperforming general-purpose vision-language models in that study [14]. More broadly, task-specific deep learning systems have already demonstrated clinically meaningful value for selected chest radiograph abnormalities in controlled evaluations [1,15].

The diagnosis-specific pattern in our study also parallels recent literature. Gains were most evident for COPD, pulmonary edema, foreign body identification, pneumothorax, cardiomegaly, and pleural effusion - abnormalities that may present with relatively conspicuous radiographic patterns. In a recent evaluation of ChatGPT-4o on chest and abdominal radiographs, higher detection rates were likewise reported for pulmonary edema, pleural effusion, cardiomegaly, emphysema, and tumor, whereas performance was weaker for pneumothorax and rib fractures [16]. The persistent failure to identify rib fractures in our cohort is therefore notable but not unexpected because rib-fracture detection on chest radiography is well recognized as difficult and unreliable even outside LLM contexts [17]. The decline in atelectasis accuracy also suggests that additional context may sometimes introduce overgeneralization or misallocation of attention rather than uniformly improving interpretation.

These findings should be interpreted cautiously. Contemporary reviews describe multimodal LLMs as promising for report generation, workflow support, education, and patient communication, but they also emphasize persistent concerns regarding hallucination, limited multimodal integration, inconsistent evaluation standards, and unresolved ethical or regulatory issues [7,8]. Our results fit that broader literature: structured context improved performance, but the model remained insufficiently reliable for autonomous chest radiograph interpretation. At present, such systems appear better suited to supervised educational use, draft generation, or hypothesis generation than to unsupervised clinical deployment [7-9].

This study has several limitations. First, the sample size was small, with 50 test images distributed across 10 diagnoses, limiting generalizability. Second, the same test images were used before and after the intervention, creating the possibility of carryover effects. Third, the scoring system incorporated partial credit, which may introduce subjectivity unless applied with a highly consistent rubric. Fourth, the structured-context condition combined two components - teaching-example exposure and a structured prompting framework - so that the relative contribution of each component cannot be isolated. These design constraints are particularly important in prompt-based research, where reproducibility can be affected by prompt brittleness, stochastic model behavior, and incomplete reporting of optimization procedures [9,10]. Future studies should evaluate these elements separately, use larger and more diverse datasets, incorporate more explicit scoring rubrics, and include external validation.

## Conclusions

Structured contextual guidance improved multimodal LLM performance on selected chest radiograph tasks, but overall accuracy remained insufficient for independent diagnostic use. The gains observed in this pilot study appear to reflect the effect of contextual scaffolding rather than durable model retraining. Future work should use larger datasets, standardized scoring methods, external validation, and more precise analyses of which prompting components contribute most to improved performance.
